# High-capacity free-space optical communications using wavelength- and mode-division-multiplexing in the mid-infrared region

**DOI:** 10.1038/s41467-022-35327-w

**Published:** 2022-12-10

**Authors:** Kaiheng Zou, Kai Pang, Hao Song, Jintao Fan, Zhe Zhao, Haoqian Song, Runzhou Zhang, Huibin Zhou, Amir Minoofar, Cong Liu, Xinzhou Su, Nanzhe Hu, Andrew McClung, Mahsa Torfeh, Amir Arbabi, Moshe Tur, Alan E. Willner

**Affiliations:** 1 Department of Electrical Engineering, University of Southern California, Los Angeles, CA 90089 USA; 2 Ultrafast Laser Laboratory, College of Precision Instrument and Optoelectronics Engineering, Tianjin University, 300072 Tianjin, China; 3 Department of Electrical and Computer Engineering, University of Massachusetts Amherst, Amherst, MA 01003 USA; 4 School of Electrical Engineering, Tel Aviv University, Ramat Aviv, 69978 Israel; 5 Dornsife Department of Physics & Astronomy, University of Southern California, Los Angeles, CA 90089 USA

**Keywords:** Fibre optics and optical communications, Mid-infrared photonics

## Abstract

Due to its absorption properties in atmosphere, the mid-infrared (mid-IR) region has gained interest for its potential to provide high data capacity in free-space optical (FSO) communications. Here, we experimentally demonstrate wavelength-division-multiplexing (WDM) and mode-division-multiplexing (MDM) in a ~0.5 m mid-IR FSO link. We multiplex three ~3.4 μm wavelengths (3.396 μm, 3.397 μm, and 3.398 μm) on a single polarization, with each wavelength carrying two orbital-angular-momentum (OAM) beams. As each beam carries 50-Gbit/s quadrature-phase-shift-keying data, a total capacity of 300 Gbit/s is achieved. The WDM channels are generated and detected in the near-IR (C-band). They are converted to mid-IR and converted back to C-band through the difference frequency generation nonlinear processes. We estimate that the system penalties at a bit error rate near the forward error correction threshold include the following: (i) the wavelength conversions induce ~2 dB optical signal-to-noise ratio (OSNR) penalty, (ii) WDM induces ~1 dB OSNR penalty, and (iii) MDM induces ~0.5 dB OSNR penalty. These results show the potential of using multiplexing to achieve a ~30X increase in data capacity for a mid-IR FSO link.

## Abstract (web summary)

A 300-Gbit/s free-space optical communication system is demonstrated in the mid-IR wavelength region by using both wavelength- and mode-division multiplexing.

## Introduction

There is growing interest in the mid-IR region for potential applications in communications, sensing, and imaging in both free space and fiber^1–3^. For free-space optical communication links, the mid-IR has several transmission windows that provide a relatively low atmospheric absorption in comparison to the C-band (1530–1565 nm)^4^. For example, it was reported that 3–5 μm and C-band wavelengths could have ~7% and ~11% atmospheric attenuation after 2 km sea-level horizontal free-space propagation with clear weather, respectively^5^. Moreover, mid-IR wavelengths tend to have better penetration through inclement weather conditions^6^. For example, it was reported that 3–5 μm and C-band wavelengths could have ~24% and ~40% atmospheric attenuation after 2 km sea-level horizontal free-space propagation with hazy weather conditions, respectively^5^. There have been reports of mid-IR communication systems that have used native mid-IR transmitters/receivers (e.g., quantum cascade lasers) to achieve up to 11-Gbit/s data rate using direct detection^7–10^. In these systems without wavelength conversion, the transmitter modulates data directly onto the mid-IR wavelengths and the receiver directly detects the data-carrying mid-IR wavelengths to recover the data^7–10^. There have also been reports of mid-IR communication systems that have used C-band devices and wavelength conversions^11–13^ to achieve 10 Gbit/s quadrature-phase-shift keying (QPSK) using coherent detection^12,13^. At the transmitter side, the data channels are modulated onto the C-band wavelengths, and these data-carrying wavelengths are converted to the mid-IR wavelengths by wavelength conversion. At the receiver side, the data-carrying mid-IR wavelengths are wavelength converted to the C-band, and these wavelengths are detected by the C-band receiver to recover the data^11–13^. Importantly, these transmission achievements have employed only a single data channel on a single beam.

Similar to communications in optical and radio systems, multiplexing multiple independent data channels and transmitting them simultaneously has produced dramatic capacity increases. Key examples are frequency- and wavelength-division multiplexing in RF and optical systems, in which each channel occupies a different frequency or wavelength^14–16^. Specifically, wavelength-division-multiplexing (WDM) has been ubiquitously deployed in the conventional C-band wavelength^15,16^. However, to the best of our knowledge, we are not aware of reports demonstrating multiple-channel WDM transmission in the mid-IR.

Another capacity-increasing multiplexing technique is space-division-multiplexing (SDM)^17,18^. One form of SDM is mode-division-multiplexing (MDM), in which multiple independent data channels are simultaneously transmitted on different beams each located on an orthogonal spatial mode^18^. For example, one modal basis set includes orbital-angular-momentum (OAM) modes, which is a subset of Laguerre-Gaussian (LG) modes^19^. A beam that carries OAM (i) has a phasefront that “twists” in a helical fashion as it propagates, (ii) has a central intensity null, and (iii) can be characterized by the OAM order *ℓ*, which is the number of 2*π* phase shifts in the azimuthal direction. Although there have been demonstrations of generating and detecting an OAM beam^20^, to the best of our knowledge, we are not aware of reports demonstrating multiple-channel MDM transmission in the mid-IR.

In this article, we experimentally demonstrate a mid-IR FSO communication system using WDM, MDM, and a combination of WDM and MDM. As the proof-of-principle experiment, we demonstrate the multiplexing of multiple 50 Gbit/s QPSK channels in only a single domain as follows: (i) WDM only: three channels with different wavelengths on a single polarization near 3.4 μm (3.396, 3.397, and 3.398 μm) each carried by a single Gaussian beam and (ii) MDM only: two channels sent on two different OAM beams (+1 and +3) at a single mid-IR wavelength and on a single polarization. In addition, we show the compatibility of these individual multiplexing approaches and demonstrate WDM + MDM by transmitting six data channels on a single polarization located at three wavelengths with each carrying two beams on different modes. The operation is as follows: (i) at the transmitter, a C-band Gaussian beam is QPSK modulated, wavelength converted to the mid-IR by difference frequency generation (DFG)^11–13^ and then converted into an OAM beam by a spiral phase plate (SPP); and (ii) at the receiver, converting the OAM back to a Gaussian beam using a SPP and wavelength converting into the C-band using DFG^11–13^. According to the BER results, the OSNR penalties at the forward error correction (FEC) threshold are estimated to be: (i) ~2 dB for wavelength conversion; (ii) ~1 dB for wavelength multiplexing; and (iii) ~0.5 dB for inter-modal crosstalk. Compared with previous demonstrations of data-carrying WDM systems and OAM-based MDM systems^15–19^, we explore the data transmission/detection in the mid-IR wavelength region using the scenarios of WDM only, OAM-based MDM only, and a combination of both multiplexing techniques. By utilizing both multiplexing techniques and the wavelength conversions between the C-band and the mid-IR, we achieve the multiplexing of six data channels with a total capacity of 300 Gbit/s, corresponding to a ~30× increase in comparison to previous single-channel, single-beam mid-IR demonstrations^7–12^.

We note that this article is an extension of our previous conference paper^21^, and this article includes: (i) results and analysis for the generated mid-IR power with different PPLN temperatures and signal wavelengths to show the optimization of wavelength conversion efficiency, and (ii) BERs of the fundamental-Gaussian-based WDM channels to show the performance of a mid-IR WDM-only system.

## Results

### Concept of mid-IR FSO communication system using both WDM and MDM

**Fig. 1:**
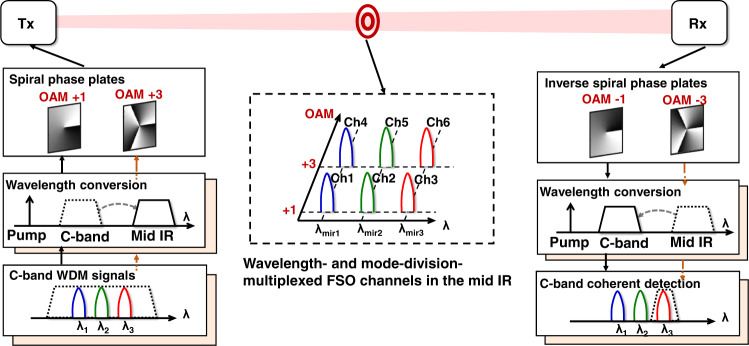
Concept for the mid-infrared (IR) wavelength-division-multiplexing (WDM) and orbital angular momentum (OAM)-based mode-division-multiplexing (MDM) free-space optical (FSO) communication system. Mid-IR WDM signals are generated by wavelength converting C-band signals using the difference-frequency generation (DFG) process, and detected at the C-band after being converted via another DFG process. Mid-IR OAM beams are generated by passing mid-IR Gaussian beams through spiral phase plates (SPPs), and converted back to Gaussian beams using SPPs of inverse orders.

The conceptual diagram of the mid-IR WDM and MDM FSO communication system is shown in Fig. 1. Multiple data-carrying beams with different mid-IR wavelengths and orthogonal OAM modes are multiplexed and co-propagate through free space. To use the widely available C-band transceivers, wavelength conversions between the C-band and mid-IR are performed in the nonlinear devices. At the transmitter, a Gaussian beam at the C-band is modulated with QPSK data and wavelength converted by a periodically-poled lithium niobate (PPLN) waveguide, where the second-order susceptibility, *χ*^(2)^_,_ results in a three-wavelength mixing process. Specifically, the wave mixing process involves the interaction of three wavelengths, including the C-band signal wavelength (*λ*_signal_), a pump wavelength (*λ*_pump_), and an idler wavelength (*λ*_idler_)^11–13^. The idler wavelength can be generated at the difference frequency and can be calculated as follows:^11–13^

1 $$1/{\lambda }_{idler}=1/{\lambda }_{pump}-1/{\lambda }_{signal}$$

Thus, mixing a signal at ~1550 nm with a pump at 1064 nm results in an idler wavelength of ~3400 nm. Moreover, if the WDM channels are all in the phase-matching bandwidth of the PPLN waveguide, they can be simultaneously converted in the same PPLN waveguide. In addition to the wavelength degree of freedom, represented by WDM, OAM multiplexing is used to further increase data capacity. Mid-IR OAM beams are generated by passing the fundamental Gaussian beam through SPPs with different orders. At the receiver, an SPP with an inverse order is used to convert the corresponding OAM channel back to the Gaussian beam for signal detection and data recovery. Subsequently, the mid-IR Gaussian beam is mixed with a 1064-nm pump. A similar DFG process in another PPLN waveguide converts the mid-IR idlers to the C-band signals, with the wavelengths calculated as follows:^11–13^

2 $$1/{\lambda }_{signal}=1/{\lambda }_{pump}-1/{\lambda }_{idler}$$

In our experiment, the pump laser is split into two paths and used for the transmitter and receiver. However, in a typical communication scenario, two different pump lasers are used at the transmitter and receiver. The different pump lasers could have a phase difference, frequency difference, and a power difference, which can potentially impact the system as follows. (i) The phase difference between the pump lasers adds phase noise to the recovered C-band signal at the receiver and the variance of the additional phase noise is proportional to the pump linewidth;^22^ (ii) The frequency difference causes a frequency shift between the generated C-band signal at the transmitter and the recovered C-band signal at the receiver, which also causes different phase-matching conditions for the wavelength conversions in the transmitter and receiver; (iii) The power difference might cause different conversion efficiencies between the transmitter and the receiver. Moreover, the phase noise and frequency shift caused by the different pump lasers could be potentially compensated by the digital signal processing (DSP) at the receiver.

### Wavelength conversions between the C-band and mid-IR wavelengths

**Fig. 2:**
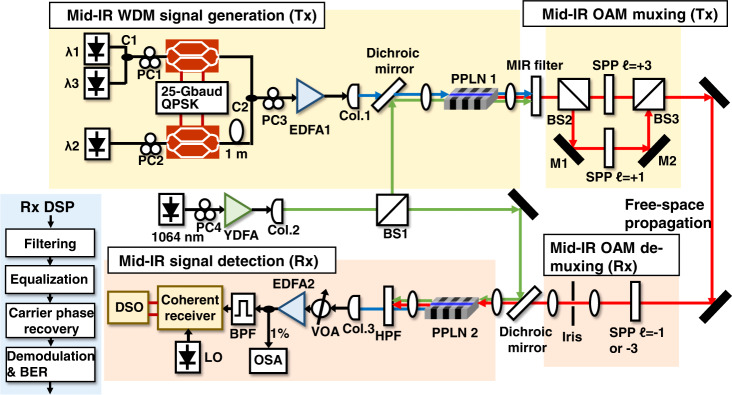
Experimental setup of the free-space mid-infrared WDM and MDM communication system. At the transmitter, C-band WDM signals are combined with a 1064 nm pump and coupled into a PPLN waveguide. The generated mid-IR beam is split into two paths and transmitted through different SPPs to generate two OAM beams. At the receiver, an SPP with an inverse OAM order is used to convert one of the OAM beams back to the fundamental Gaussian beam. The converted beam is combined with the 1064 nm pump and coupled into another PPLN waveguide. Finally, the generated C-band signal is detected and processed by a coherent receiver. PC: polarization controller, Col.: collimator, EDFA: erbium-doped fiber amplifier, YDFA: ytterbium-doped fiber amplifier, PPLN: periodically poled lithium niobate, M: mirror, SPP: spiral phase plate, HPF: high-pass filter, BPF: tunable band-pass filter, LO: local oscillator. VOA: variable optical attenuator, OSA: optical spectrum analyzer, DSO: digital storage oscilloscope, C: fiber-based optical coupler, BS: free-space beam splitter.

**Fig. 3:**
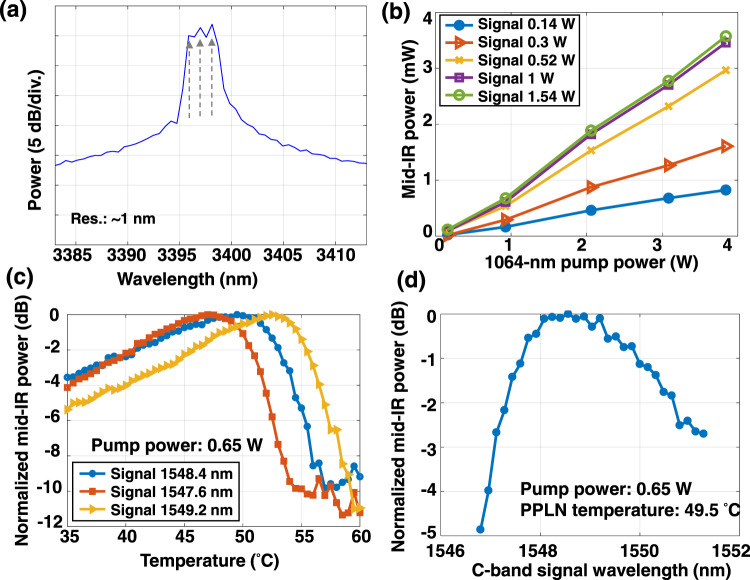
The generation of mid-IR WDM signals through the difference-frequency generation process in the periodically poled lithium niobate (PPLN) waveguide. **a** Spectrum of the generated mid-IR WDM signals with a resolution of ~1 nm. Arrows indicate the three mid-IR WDM channels. **b** Generated mid-IR beam power as a function of the 1064 nm pump power with different signal power values. The C-band signal wavelength is set at 1550 nm. **c** Generated mid-IR beam power as a function of PPLN temperature with different C-band signal wavelengths. **d** Generated mid-IR beam power as a function of C-band signal wavelength with a PPLN temperature of 49.5 °C.

We experimentally demonstrate a mid-IR WDM and OAM-multiplexed FSO communication link using the setup illustrated in Fig. 2 (for a detailed description, see the Methods section). First, we measure the link performance of a WDM system with the mid-IR Gaussian beam. As shown in the spectrum depicted in Fig. 3(a), three WDM channels at the ~3400 nm wavelength (3.396, 3.397, and 3.398 μm) with a channel spacing of 27.5 GHz (~1 nm @ 3400 nm) are generated through the DFG process. We set the channel spacing of 27.5 GHz by tuning the frequency of the three C-band lasers used at the transmitter. However, the spectral shapes of the channels are not completely resolved by the optical spectrum analyzer (OSA) for the mid-IR, as the OSA has a limited resolution bandwidth of 1 nm which is wider than the data channels themselves. The pump and C-band signal power, as well as the PPLN1 temperature control, are adjusted to optimize the conversion efficiency of the mid-IR beam generation. Figure 3(b) shows the power of the generated mid-IR beam as a function of the pump power for different signal power values. The PPLN1 temperature is 49.5 °C for Fig. 3(b). The mid-IR power generally increases with the pump power. It also increases with the signal power but tends to saturate at signal power levels higher than 1 W. This might be due to an imbalance between the signal and pump photon numbers, possibly resulting in pump depletion^23^. Figure 3(c) shows the mid-IR power as a function of the PPLN temperature for three C-band signal wavelengths. The mid-IR power is measured by a free-space power meter with a sensor covering 0.19–20 μm wavelengths. The PPLN1 temperature is adjusted to satisfy the quasi-phase-matching in the DFG process. To determine the temperature that gives the optimal conversion efficiency for 1548.4 nm, the PPLN temperature is tuned by a temperature controller and the conversion efficiency is measured accordingly. As shown in Fig. 3(c), the optimal temperature is 49.5 °C for 1548.4 nm. When the C-band signal wavelength is longer, the optimal temperature tends to increase. When the pump power is 3.86 W and the PPLN1 temperature is 49.5 °C, the conversion efficiency is ~−26.5 dB, which is the ratio between the output idler power and the input signal power of the PPLN1. The conversion efficiency could decrease when the pump power is lower or the PPLN temperature is not optimal. The fact that there are different optimal temperatures for different C-band signal wavelengths could affect the conversion efficiency of the WDM channels. Figure 3(d) shows the generated mid-IR power as a function of the C-band signal wavelength when the PPLN temperature is set at 49.5 °C. The pump power at the PPLN1 input is ~0.65 W for Fig. 3(c, d). We note that the generated mid-IR power shown in Fig. 3(c) is normalized for each C-band signal wavelength; however, the generated mid-IR power in Fig. 3(d) is normalized for a fixed temperature value of 49.5 °C. Therefore, the normalized power values in Fig. 3(c) and (d) are not directly comparable. At a ~1.6 nm C-band signal wavelength bandwidth, the generated mid-IR power is >90% of the maximum generated mid-IR power. This allows for simultaneous wavelength conversion of a few WDM channels in a single PPLN waveguide. The wavelength conversion from the mid-IR to the C-band is performed in PPLN2. The total input power of all 3 mid-IR channels into PPLN2 is 0.956 mW, and the output power of all C-band channels is 0.0053 mW.

### Mid-IR FSO communication system using WDM only

**Fig. 4:**
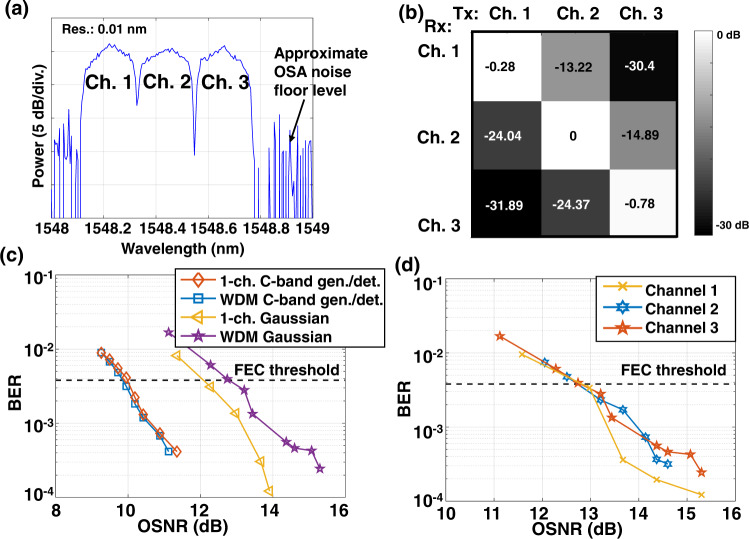
Demonstration of a three-channel mid-IR WDM FSO communication system with the Gaussian beam. **a** Spectrum of the WDM signals that are converted back to the C-band. **b** Normalized optical crosstalk matrix of WDM. **c** Measured bit error rate (BER) as a function of the received optical signal-to-noise ratio (OSNR) for a C-band generation/detection (gen./det.) and mid-IR Gaussian beam transmission. In the C-band generation/detection case, C-band signals are detected by the coherent receiver without wavelength conversion and free-space propagation. **d** Measured BER as a function of the received OSNR for the three mid-IR WDM channels.

Next, we demonstrate a 150 Gbit/s mid-IR WDM FSO communication system with the Gaussian beam. Figure 4(a) shows the spectrum of the recovered signal at the C-band after PPLN2, in which three WDM channels and an approximate OSA noise floor level can be seen. The C-band wavelengths shown in Fig. 4(a) are chosen such that the central channel wavelength is the optimal C-band signal wavelength in Fig. 3(d) with the highest generated mid-IR power. A normalized crosstalk matrix of the WDM channels is shown in Fig. 4(b). The crosstalk matrix is obtained by sending different single wavelength channels modulated with a 25 Gbuad QPSK signal at the transmitter and measuring the output optical power of a tunable band-pass filter with different center wavelengths at the receiver. The crosstalk tends to be larger from the longer- to shorter-wavelength channels than from the shorter- to longer-wavelength channels. This might be due to the optical filter used in the measurement, which has a higher extinction ratio at the shorter wavelengths than at the longer wavelengths. The measured WDM crosstalk matrix shows crosstalk lower than −13 dB between adjacent wavelengths. We note that the WDM crosstalk critically depends on the sharpness of the optical filter, and it can be suppressed by further DSP after coherent detection.

Subsequently, 25 Gbaud QPSK signals are transmitted on each WDM channel. In the C-band generation/detection case, the signal generated by the C-band transmitter is directly received by the coherent receiver without wavelength conversion and free-space propagation. As shown in Fig. 4(c), the mid-IR cases have OSNR penalties compared to C-band generation/detection cases. These OSNR penalties might be caused by the wavelength conversions where (i) undesired terms might be generated by the PPLN and overlap with the mid-IR data channels leading to in-band crosstalk and (ii) additional frequency drift and phase noise from the pump laser might be added to the mid-IR data channels^24^. In both the single-channel and WDM cases, only mid-IR Gaussian beams are used, and the SPPs are bypassed. The received signal is coupled to a single-mode fiber and amplified by an erbium-doped fiber amplifier (EDFA) to compensate for the power loss. A variable optical attenuator (VOA) is used before the EDFA to change EDFA input power and the OSNR of the EDFA output. A C-band OSA is used to measure the OSNR of the signal. For the BER measurement, the signal into the coherent receiver has similar power and different OSNR values. This is achieved by (i) changing the EDFA input power by the VOA, (ii) changing the gain of the EDFA, and (iii) setting the received data channel power into the coherent receiver to 3 dBm. The noise figure of the EDFA is <4 dB. At the receiver, we use an EDFA to amplify the received signal, where the amplified spontaneous emission noise might be added. If the received signal power changes due to the free-space link loss, the OSNR of the EDFA output might change accordingly. We choose the BER-OSNR curve as a figure of merit to investigate the penalties caused by the wavelength conversion and crosstalk between multiplexed channels^15^. It might also be possible to use the BER vs. received mid-IR power as a figure of merit^11^. We find that the single-channel mid-IR Gaussian beam has a ~2 dB OSNR penalty at the BER near the FEC threshold of 7% overhead in comparison to the C-band generation/detection case. To help suppress crosstalk from adjacent channels, we use digital filtering in the receiver after coherent detection^25^. The mid-IR WDM channel has an additional ~1 dB OSNR penalty compared to the mid-IR single-channel transmission. We note that some of the measured power penalty might be due to crosstalk induced by the wavelength conversion in which undesired mixing terms might be generated by the PPLN and interfere with the mid-IR data channels^24^. Figure 4(d) shows the measured BERs as a function of the received OSNR for all three WDM channels. We note that the BER vs. OSNR curves in Fig. 4(c, d) are not completely smooth (i.e., there is some “wiggle” in the measurements). This could be due to measurement error of OSNR value, which might be induced by the power measurement error of the OSA.

### Mid-IR FSO communication system using MDM and a combination of WDM and MDM

**Fig. 5:**
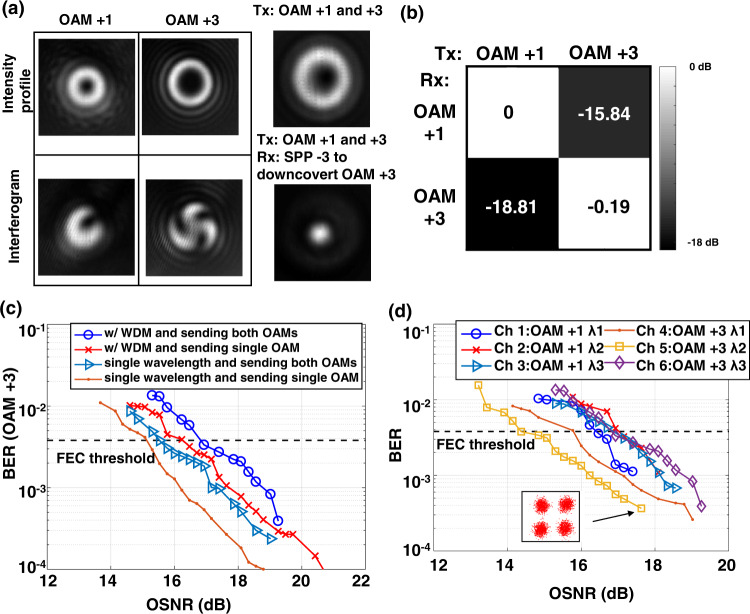
Demonstration of a 300 Gbit/s mid-IR FSO communication system using WDM and a combination of WDM and MDM. **a** Measured beam profile of the mid-IR OAM beams. Intensity profile and interferogram with a Gaussian beam of the OAM + 1 and OAM + 3 beam, respectively. Intensity profile of the data-carrying multiplexed OAM + 1 and +3 beams. Intensity profile of the multiplexed OAM beam after passing through the second SPP with OAM order −3. **b** Normalized crosstalk matrix of MDM. **c** Measured BER of the OAM + 3 channel as a function of the received OSNR for the mid-IR OAM beam transmission when sending both OAM modes and sending a single OAM mode. **d** Measured BER and OSNR of all the channels, including two OAM modes with three wavelengths on each mode.

Subsequently, we demonstrate a mid-IR FSO communication system using OAM-multiplexing and a combination of WDM and OAM-multiplexing with up to 300 Gbit/s capacity. Figure 5(a) shows the experimentally measured beam profiles of the generated 3.4 μm OAM beams. The intensity profiles of the OAM + 1 and OAM + 3 beams are shown in Fig. 5(a) on the top row, respectively. The intensity profiles are ring-shaped due to the phase singularity at the center^26^. To verify the OAM orders of the generated beams, interferograms of the OAM beams with a Gaussian beam are captured and shown in Fig. 5(a) on the bottom row. The interferograms have twisted arms, and the number of twists corresponds to the OAM order of the beam. When two data-carrying OAM + 1 and +3 beams are multiplexed, the intensity profile has a ring shape, as shown in Fig. 5(a) on the top right. The intensity profile when the multiplexed OAM beams pass through the inverse SPP with an OAM order of −3 is shown in Fig. 5(a) on the bottom right. The intensity profile has a Gaussian-like beam at the center, which corresponds to the OAM + 3 beam. The other OAM + 1 beam still has a ring shape after the inversed SPP. This intensity profile indicates that the desired OAM beam can be converted to the fundamental Gaussian mode and separated from the other beam with a spatial filter.

Figure 5(b) shows a normalized crosstalk matrix of the OAM multiplexed channels. The values in the crosstalk matrix indicate the measured optical power that is converted back to the C-band coherent receiver when a single mid-IR OAM beam is transmitted, and an inversed SPP is used to receive one OAM mode. The residual crosstalk between the OAM channels could be caused by the misalignment between the mid-IR beam axis and the center of the SPPs, which may degrade the quality of the generated OAM beams and the back-converted Gaussian beams.

Figure 5(c) shows the measured BERs as a function of the received OSNR for the single OAM beam transmission and two multiplexed OAM beams. As shown by the BER curves, the OAM multiplexing induces a < 1 dB OSNR penalty at the FEC threshold. This could be caused by modal crosstalk between the OAM channels. In addition, the WDM induces a ~1 dB OSNR penalty compared to the single-wavelength case. This could be due to the distortion caused by wavelength conversions.

In this demonstration, two OAM beams each contain three wavelength channels that are multiplexed, resulting in a total of six channels. The BER performance of the six channels is shown in Fig. 5(d). We receive one channel at a time in the proof-of-concept experiment. Considering a real communication system (M modes and N wavelengths), M×N parallel transmitters/receivers would be required to transmit/receive independent data streams. Each transmitter has a laser source and an IQ modulator, and each coherent receiver has a local oscillator laser. Moreover, at the transmitter, M PPLNs would be used to convert the wavelength of the signals, each with a mid-IR filter at the output. At the receiver, another M PPLNs would be used to convert the mid-IR wavelengths. In our experiment, SPPs and beam splitters are used to convert and combine the OAM beams. The MDM could also be potentially realized by other methods, which might be more efficient and more compact, such as multi-plane light conversion^27^ and Dammann gratings^28^. However, special designs might be required for them to work in the mid-IR region. The channels have slightly different performances, which could be due to the different crosstalk values of each channel. For all the channels, BER below the 7% FEC threshold can be achieved. This indicates that a total gross data capacity of 300 Gbit/s is transmitted through the mid-IR FSO communication system.

## Discussion

This paper explores the use of WDM and MDM multiplexing techniques to increase data capacity in mid-IR communications. A few points worth discussing include:

(i) In our approach, wavelength conversion is utilized to convert the signals between the C-band and mid-IR, such that widely available, high-performance C-band components can be used to enable high-speed data generation and detection. Although many native mid-IR devices are available (e.g., narrow-linewidth lasers^29,30^ and optical amplifiers^31^), high-speed mid-IR modulators and photodetectors are still not easily found but can be used when available.

(ii) In our demonstration, we multiplex three wavelength channels, which might be mainly limited by the PPLN phase-matching bandwidth. To scale the number of wavelength channels in our scheme, a nonlinear device with a wider phase-matching bandwidth might be required. Recently, mid-IR generation in nonlinear devices with wide phase-matching bandwidth have been reported (e.g., 700 nm phase-matching bandwidth of the thin-film lithium niobate by dispersion engineering^32^).

(iii) Our work uses a high level of optical power to pump the PPLN waveguides due to the relatively low conversion efficiency of ~−26.5 dB (<0.2%). However, the power requirement can be potentially reduced when more efficient PPLN waveguides are available (e.g., 3.1% conversion efficiency for mid-IR generation^33^).

(iv) The data channel mid-IR wavelength depends on the pump wavelength and nonlinear device. Although we worked at ~3.4 μm, other wavelengths can be demonstrated by judiciously using other appropriate pump wavelengths and nonlinear devices^11^.

(v) In general, polarization-division-multiplexing (PDM) is potentially compatible with WDM and MDM, thereby offering another avenue for capacity increase^15^. In our current experimental setup, all channels are at a single polarization using the single-polarization IQ modulators at the Tx and polarization-sensitive PPLNs at the Tx and Rx. We believe that PDM can potentially be implemented in our approach, and some techniques that might help achieve this include: (a) Modulator: a dual-polarization IQ modulator to generate PDM signals^34^, and (b) PPLN: polarization diversity PPLN architecture to perform the wavelength conversions of the polarization-multiplexed signals^35^.

(vi) The results described in this paper are for free-space communications. However, many of the same principles for the transmitter and receiver should still be valid in an optical fiber communication system, and there is interest in low-loss fibers for mid-IR wavelengths^1^.

(vii) We wish to mention that eye safety is, in general, an important issue for FSO communication systems^36^. As one example under specific values of transmitter aperture diameter and exposure time, an eye safety standards document states that the total transmitted power should be <10 mW for wavelengths >1.4 μm^37^. We recommend consulting such standards documents when designing a mid-IR free-space communication system, such that the total power in the M×N data-carrying beams (i.e., M modes on each of N wavelengths) should be less than the recommended eye safety value^36,37^. For our demonstration, each of the six beams is ~0.3 mW, which we believe could be designed in the future to be within eye safety recommendations.

(viii) Our proof-of-principle demonstration is for a ~0.5 m free-space propagation distance in a lab environment without considering the potential channel impairments. However, an FSO link in the mid-IR could be affected by various factors that can degrade system performance, especially under longer distances, including the following:

(1) The received signal could be attenuated due to atmospheric absorption by water vapor or other molecules^5^. This attenuation induces loss on the received mid-IR signal power and can degrade the OSNR and measured BER^38^. For comparison, many previous FSO demonstrations have used C-band wavelengths^15–19^, yet the 3–5 μm wavelength range could have a lower atmospheric attenuation (e.g., ~7% and ~11% attenuation after ~2 km sea-level horizontal propagation for ~3.4 μm and ~1.55 μm wavelengths under clear weather, respectively^5^). Such lower atmospheric attenuation in the mid-IR might result in a higher received signal power and a lower BER given the same link distance and transmitted signal power^38^.

(2) In general, atmospheric turbulence can cause random phasefront distortions on the transmitted beam^39^, resulting in scintillation^11^ and beam wandering^40^ that leads to power loss of the received signal^11^. The influence of phasefront distortions tends to be larger for shorter wavelengths, longer propagation distances, and higher turbulence strengths (e.g., C_n_^2^ value, which is relatively wavelength independent when considering 1.55–3.4 μm wavelengths)^11,39^.

(a) For a Gaussian-beam, the mid-IR could possibly have lower turbulence-induced effects than the C-band because the phase distortion is potentially weaker for the mid-IR given the smaller phase delay for a certain propagation distance^11,39^. Such distortion can reduce the received signal power and increase the BER.

(b) For an OAM-based MDM link, the turbulence-induced phasefront distortion might additionally lead to inter-channel crosstalk. For example, we simulate turbulence effects on mid-IR (3.4 μm) and C-band (1.55 μm) OAM beams; please see the Supplementary Material for more detail that helps provide some context and support for the following statements. Our simulation results show that for an OAM + 3 beam over a 2 km propagation distance under turbulence strengths of C_n_^2^ = 1 × 10^−16^ m^−2/3^ (weaker) and C_n_^2^ = 1×10^−14^ m^−2/3^ (stronger)^39^, the 3.4 μm wavelength tends to have ~6.8 dB and ~6.4 dB lower modal power coupling, respectively, than the 1.55 μm wavelength. The modal coupling can produce inter-channel crosstalk and a system penalty in an OAM-based MDM system.

(3) In general, the beam divergence depends on various parameters, including wavelength, propagation distance, and mode order^41^. Due to the larger beam divergence of the mid-IR wavelength compared to the C-band wavelength, a larger receiver aperture might be required for a given link distance in order to recover enough signal power and reduce the BER. In addition:

(a) For a Gaussian-based link, a limited-size receiver aperture could truncate the received beam. As an example, we consider a Gaussian beam with a 10 cm beam radius at the transmitter. From a simple calculation based on basic beam propagation formulae^41^, the beam radius grows to ~10.05 cm and ~10.23 cm for the 1.55 μm and 3.4 μm wavelengths, respectively, after 2 km distance propagation. When considering a 10 cm receiver aperture radius, the power loss due to divergence would be ~0.6 dB and ~0.7 dB for the 1.55 μm and 3.4 μm wavelengths, respectively.

(b) For an OAM-based MDM link, the limited-size receiver aperture might additionally induce modal power coupling from the desired mode to other modes, especially when considering that an LG beam has two indices, azimuthal (*ℓ*) and radial (*p*). Indeed, beam truncation can cause coupling to other p mode values, thereby inducing loss on an OAM beam of a specific *ℓ* and *p* value^42^. As an example, we consider an OAM + 3 beam with a 10-cm beam radius at the transmitter. From a simple calculation^41^, the beam radius grows to ~10.8 cm and ~13 cm for the 1.55 μm and 3.4 μm wavelengths, respectively, after 2 km propagation distance. A receiver aperture radius of 10 cm would induce ~0.39 dB and ~1.7 dB loss to the OAM beam of a specific *ℓ*,*p* value of 3.0 for the 1.55 μm and 3.4 μm wavelengths, respectively. This degradation grows with higher-order OAM values^42^.

(4) In general, the pointing error might cause both angular tip/tilt and lateral displacement of the received beam^42–45^. There have been reports that investigated C-band FSO system performance under different pointing error^42–45^, and we believe mid-IR FSO systems could potentially share similar behavior^43^. Specifically:

(a) For a Gaussian-based system, the pointing error could reduce the received signal power and increase the BER as the receiver aperture might fail to capture the beam profile^43^. For example, a pointing error <13.5 μrad can achieve a 10^−9^ BER for a C-band 1 Gb/s 1 km FSO link under clear weather with 320 mW transmitted power and 5 cm Tx/Rx aperture radius^44^.

(b) For an OAM-based MDM system, the pointing error could additionally induce inter-channel crosstalk as the aperture might fail to fully capture the phase changes and induce modal power coupling^42,45^. This could require more advanced pointing accuracy. Although the conditions are somewhat different, an illustrative example of a pointing error <3 μrad transmitter pointing error could cause ~1 dB power penalty for an OAM-multiplexed system with a 1.5 cm transmitted beam radius and 2.25 cm receiver aperture radius over a 100 m propagation distance for a C-band wavelength^42^.

## Methods

### Experimental setup of the WDM MDM mid-IR FSO communication link

We generate C-band WDM channels, as shown in Fig. 2, by modulating three laser sources with two optical in-phase-quadrature modulators, each loaded with a 25 Gbaud QPSK signal. The odd and even channels are generated from different modulators. Each IQ Mach-Zehnder modulator has two electrical inputs as the in-phase and quadrature signals. The signals are fed to a nested Mach-Zehnder interferometer (MZI) structure. The continuous-wave light input is split into two copies and modulated with the two electrical signals in the MZI structure. The quadrature signal experiences a *π*/2 shift before the superposition of the two light streams, and the IQ modulator has an electro-optical bandwidth of 25 GHz. The electrical signal is generated by an arbitrary waveform generator with a 92 GSa/s sampling rate. The electrical inputs are amplified by driver amplifiers with a bandwidth of 60 GHz before driving the IQ modulator. One branch is delayed so that the adjacent wavelength channels have decorrelated signals. The data channels are amplified with an EDFA (EDFA1) and coupled into a PPLN waveguide (PPLN1) with a 1064 nm pump laser, which is amplified with an ytterbium-doped fiber amplifier (YDFA). The C-band lasers (*λ*_1_, *λ*_2_, *λ*_3_) at the transmitter have a linewidth of ~100 kHz and output power of 10 mW. The 1064 nm laser has a linewidth of ~100 kHz and output power of 120 mW. Polarization controllers are used before the EDFA1 and YDFA to adjust the polarization of the beams into the polarization-sensitive PPLN. The total input optical power of the data channels before EDFA1 is ~0.5 mW. The output optical power of the amplified data channels after the EDFA1 is adjusted ranging from 0.14 to 1.54 W. The input optical power of the pump before the YDFA is ~10 mW. The output optical power after the YDFA is adjusted ranging from 1.5 to 7.5 W. The PPLN is temperature-controlled to adjust the quasi-phase-matching frequency and optimize mixing efficiency. A mid-IR beam is generated through the DFG process. At the PPLN1 output, two Germanium windows are used as mid-IR band-pass filters to filter out the high-power pump and input signals. The generated mid-IR fundamental Gaussian beam is split into two paths and transmitted through two SPPs with OAM orders of +1 and +3. The two OAM beams are combined, with one path delayed for data decorrelation. Subsequently, the combined beams propagate co-axially for ~0.5 m in free space.

At the receiver, the OAM beams are first de-multiplexed by SPPs of the corresponding inverse order (−1 for the OAM + 1 beam and −3 for the other beam). The SPP converts the corresponding OAM input to a Gaussian beam, while input OAM beams of other orders emerge as ring-shaped, center-null beams to be blocked by an appropriate spatial filter. The mid-IR Gaussian beam and a 1064 nm pump laser are coupled into PPLN2, which converts the mid-IR beam back to the C-band through the DFG process. At the PPLN2 output, a 1500 nm high-pass filter is used to filter out the high-power pump. The received signal is then coupled to a single-mode fiber and detected and processed by a C-band coherent receiver. The signal is amplified by an EDFA and filtered by a band-pass filter (with a bandwidth of ~1 nm and a tunable central wavelength) to suppress the out-of-band noise before being detected by the coherent receiver. For the crosstalk matrix measurement in Fig. 4(b), a band-pass filter (with a bandwidth of ~0.2 nm and a tunable central wavelength) is used. The output optical power of the local oscillator laser is 10 dBm. The optical signal power of the received data channel at the input of the coherent receiver is set to 3 dBm. The coherent receiver has a bandwidth of 40 GHz. The signal detected by the coherent receiver is sampled by an oscilloscope with an 80 GSa/s sampling rate and a 33 GHz bandwidth.

In our proof-of-principle experiment, we note that all channels are simultaneously transmitted but only one channel is detected at a time. It might be possible to detect all six channels simultaneously by modifying the receiver. This could be potentially achieved by: (i) using an OAM demultiplexer to simultaneously convert the two mid-IR OAM beams to Gaussian beams;^27^ (ii) converting the wavelength of the two mid-IR Gaussian beams to C-band in two PPLNs simultaneously; (iii) demultiplexing the three wavelength channels after each PPLN output using wavelength demultiplexers; and (iv) detecting all the channels with six coherent receivers simultaneously.

### Digital signal processing for coherent detection

Offline DSP is performed at both the transmitter and receiver. At the transmitter, the QPSK signal is Nyquist pulse shaped using a raised cosine filter with a roll-off factor of 0.05. The sharp roll-off factor of 0.05 is chosen for the raised cosine filter to reduce the spectral bandwidth of the signal and the required guard band between the WDM channels^46^. The pulse-shaped signal is resampled and loaded to the arbitrary waveform generator. The arbitrary waveform generator operates at a sampling rate of 92 GSa/s. At the receiver, the signal is detected by an optical modulation analyzer (OMA), which consists of an optical coherent receiver and a digital real-time oscilloscope. The sampled waveform by the OMA is further offline processed using the vector signal analysis software^47^. The received signal is first digitally filtered with a band-pass filter to suppress the crosstalk from the adjacent wavelengths. Carrier phase recovery (CPR) is performed to compensate for the frequency offset and phase noise. A fourth power-based coarse CPR^48^ and a Kalman filtering-based fine CPR^49^ are employed to recover the carrier phase. To compensate for the linear distortions caused by the transmitter and receiver components (e.g., driver amplifiers, IQ modulators, the optical band-pass filter, coherent receiver, and the oscilloscope), a finite impulse response feed-forward equalizer with 48 taps is applied to the received signal^47^. The tap weights of the equalizer are pre-converged in the C-band generation/detection case using training sequences and kept fixed for the remaining data signals. The BER is measured through error counting.

### OAM beam generation with SPPs

In our demonstration, the Gaussian and OAM beam transformation is performed by passing the mid-IR Gaussian beams through SPPs that have a helical surface. The height gradient of the SPP surface along the azimuthal direction is calculated using the following equation^46^

3 $$\frac{\partial h}{\partial \varphi }=\frac{{{{{{\mathscr{l}}}}}}\lambda }{2\pi (n-1)}$$

where *h* is the height of the SPP surface, *φ* is the azimuthal angle, *l* is the OAM order, *λ* is the mid-IR beam wavelength of ~3.4 μm, and *n* is the refractive index of the SPP. The SPP is made of Zinc Selenide, which has low absorption and a refractive index of ~2.4 in the mid-IR wavelength range. The measured insertion loss of the SPP is ~0.45 dB, which corresponds to >90% transmission^50^. Metasurface phase masks have also been used to impart phase profiles. Fabricated in an amorphous silicon on a sapphire platform^51^, they exhibited insertion loss of ~0.91 dB, which is slightly higher than that of the Zinc Selenide SPPs used in the current demonstration.

## Supplementary information

Supplementary Information

## Data Availability

All data, theory details, simulation detail that support the findings of this study are available from the corresponding authors upon request.

## Code availability

All relevant computing codes that support the findings of this study are available from the corresponding authors upon request.
