# Supplementary material for: High-capacity free-space optical communications using wavelength- and mode-division-multiplexing in the mid-infrared region
Source: Nat Commun. 2022 Dec 10;13:7662. doi: 10.1038/s41467-022-35327-w (PMC9741622; doi:10.1038/s41467-022-35327-w)
Supplement: Supplementary file 1 — Supplementary Information [file 41467_2022_35327_MOESM1_ESM.pdf]

Supplementary Information: High-Capacity Free-Space Optical Communications Using Wavelength- and Mode-Division-Multiplexing in the Mid-Infrared Region

Kaiheng Zou[1†], Kai Pang[1†], Hao Song[1], Jintao Fan[2], Zhe Zhao[1], Haoqian Song[1], Runzhou Zhang[1], Huibin Zhou[1], Amir Minoofar[1], Cong Liu[1], Xinzhou Su[1], Nanzhe Hu[1], Andrew McClung[3], Mahsa Torfeh[3], Amir Arbabi[3], Moshe Tur[4], and Alan E. Willner[1,5]

1. Dept. of Electrical Engineering, University of Southern California, Los Angeles, CA 90089, USA
2. Ultrafast Laser Laboratory, College of Precision Instrument and Optoelectronics Engineering, Tianjin University, Tianjin 300072, China
3. Dept. of Electrical and Computer Engineering, University of Massachusetts Amherst, Amherst, MA 01003, USA
4. School of Electrical Engineering, Tel Aviv University, Ramat Aviv 69978, Israel
5. Dornsife Department of Physics & Astronomy, University of Southern California, California 90089, USA
†These authors contribute equally to this work
Email: kaihengz@usc.edu willner@usc.edu

In this Supplementary information, we use a simple simulation model[1,2] to help provide some context and support to the discussion in the Discussion section related to turbulence effects in an OAM-based MDM system. Specifically, we model the issue of modal coupling of an orbital angular momentum (OAM) beam propagating through a 2-km free-space link with different turbulence strengths in the 3.4-μm and C-band wavelength regions[1,2]. We use a simulation model that is similar to the one in references 1 and 2. The simulation is performed using MATLAB with the random phase screens and Kirchhoff-Fresnel diffraction integral-based beam propagation methods[1,4].

The reader is encouraged to see references 1-6 for more specifics about the model itself.

In our model, we simulate the atmospheric turbulence using multiple phase screens, which have random phase distributions according to the Kolmogorov turbulence theory [1,3]. The phase distribution of each phase screen $\psi(\kappa)$ is obtained according to the spectrum of fluctuations $\phi_n(\kappa)$ in the refractive index[1].

$$\psi(\kappa) = \exp(\mathrm{i}\phi_n(\kappa)) \tag{1}$$

and $\phi_n(\kappa)$ is given by[1].

$$\phi_n(\kappa) = 0.023 r_0^{-5/3} k^{-11/3} \quad (2)$$

where κ is the angular spatial frequency, $k = 2\pi/\lambda$ is the wave number, and r_0 is the Fried parameter, which is related to the atmospheric structure constant C_n^2 [5].

$$r_0 = (0.423 k^2 C_n^2 L)^{-3/5} \quad (3)$$

where L is the propagation length. The input beam is projected onto the first phase screen and propagates by a 100-m distance before the next phase screen. The propagation of the beam is simulated by using the Kirchhoff-Fresnel diffraction integral[5]. The atmospheric attenuation is applied to the beam profiles during the propagation and is set to be 0.24 dB/km and 0.15 dB/km for the 1.55-μm and 3.4-μm wavelengths, respectively[6]. By repeating this process for 50 phase screens, a link distance of up to 5 km is simulated. In our simulation, the same beam waist of 5 cm is chosen for the beams with different modes and wavelengths. The input OAM beam is generated as a Laguerre-Gaussian beam with ℓ=3 and p=0. The simulation screen size is 1 m × 1 m and the pixel size is 0.5 mm. At the receiver, a 10-cm aperture radius is considered. Each data point is calculated by averaging 50 random turbulence realizations. Examples of daytime measurements of C_n^2 near the ground can have values ranging from 10^{-13} to 10^{-14} $m^{-2/3}$, whereas C_n^2 can decrease to 10^{-16} at an altitude of 1 km[1]. Therefore, we choose C_n^2=1×10^{-16} $m^{-2/3}$ and 1×10^{-14} $m^{-2/3}$ as examples of the relatively weaker and stronger turbulence.

Figure S1 shows the received modal power distribution of different OAM modes under different turbulence strengths and propagation distances. As shown in Fig. S1(a), the turbulence strength is C_n^2=1×10^{-16} $m^{-2/3}$ and an OAM +3 beam is transmitted through the turbulence. The turbulence-induced power coupling to other azimuthal (ℓ) mode values increases with the propagation distance. The beam with a 3.4-μm wavelength has ~6.8-dB lower crosstalk to other modes in comparison to the beam with a 1.55-μm wavelength when the propagation distance is 2

km. As shown in Fig. S1(b), when the turbulence strength is C_n^2=1×10^{-14} m$^{-2/3}$, the modal power coupling to other modes becomes stronger. Similarly, the beam with a 3.4-μm wavelength shows ~6.4-dB lower modal power coupling to other modes than the beam with a 1.55-μm wavelength. The reason could be that given the same turbulence strength C_n^2, the D/r_0 increases (D = beam diameter) when the wavelength increases, i.e., the phase distortion is stronger [1]. We also note that the beam size of the 3.4-μm wavelength beam tends to grow larger than the 1.55-μm wavelength beam with the same transmitted beam waist and OAM order after 2-km propagation. With the chosen receiver aperture size, the 3.4-μm wavelength beam tends to be truncated at the 2-km distance, which causes power loss on the received specific azimuthal-radial OAM mode value.

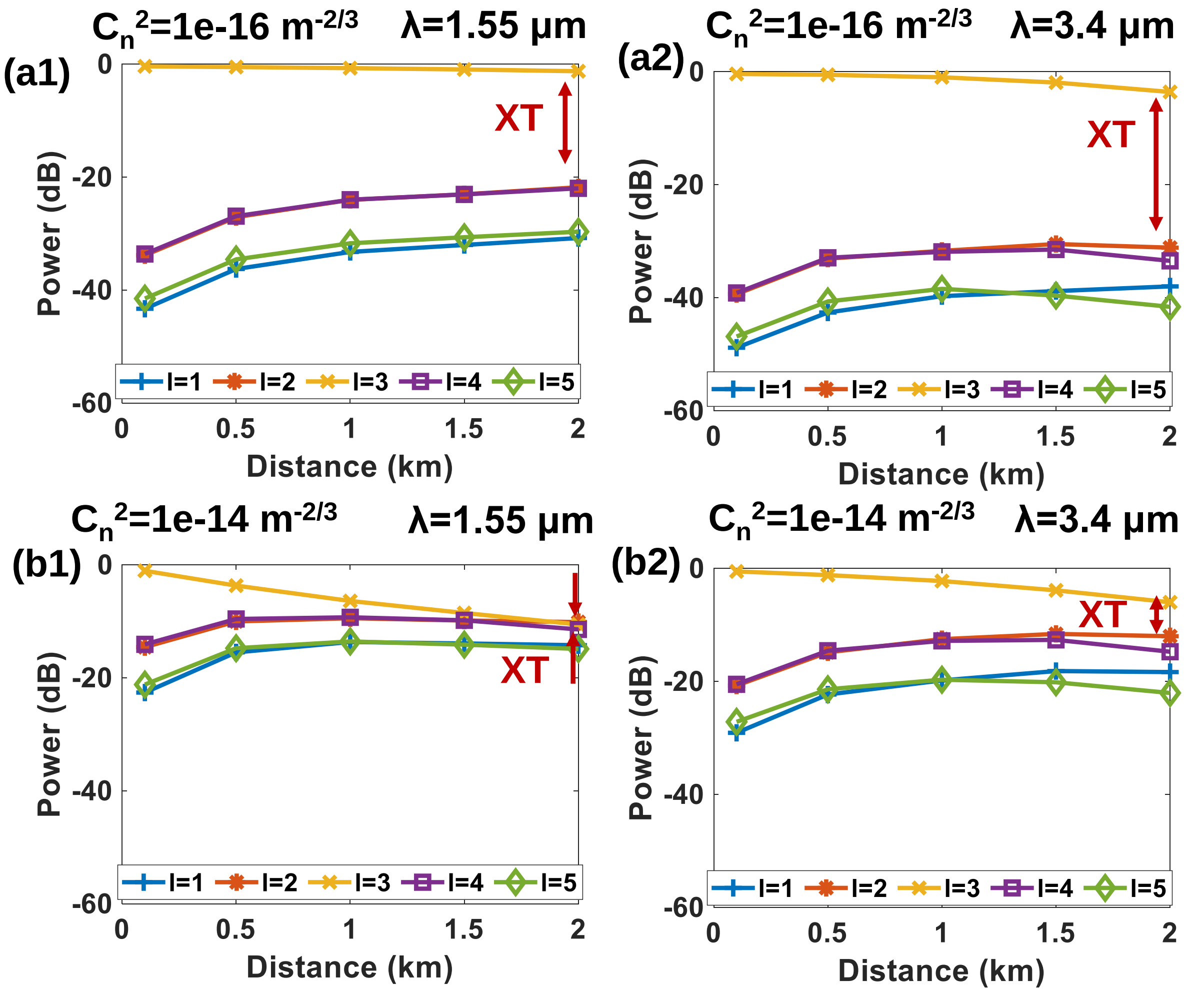

Fig. S1 The modal power distribution of the received beam at different propagation distances through turbulence with a strength of (a) C_n^2=1×10^{-16} m$^{-2/3}$ for a beam carrying OAM +3 with the wavelengths of (a1) 1.55 μm and (a2) 3.4 μm; and (b) C_n^2=1×10^{-14} m$^{-2/3}$ for a beam carrying OAM +3 with the wavelengths of (b1) 1.55 μm and (b2) 3.4 μm. XT: crosstalk.

References

1. L. C. Andrews and R. L. Phillips, *Laser Beam Propagation through Random Media* (SPIE, 2005).
2. Z. Zhao, R. Zhang, H. Song, K. Pang, A. Almaiman, H. Zhou, H. Song, C. Liu, N. Hu, X. Su, A. Minoofar, H. Sasaki, D. Lee, M. Tur, A. F. Molisch, and A. E. Willner "Modal coupling and crosstalk due to turbulence and divergence on free space THz links using multiple orbital angular momentum beams," *Sci. Rep.* **11**, 2110 (2021).
3. H. Samimi, "Distribution of the sum of K-distributed random variables and applications in free-space optical communications," *IET Optoelectron.* **6**, 1 (2012).
4. R. Edgar, "The Fresnel diffraction images of periodic structures," *J. Mod. Opt.* **16**, 281–287 (1969).
5. G. A. Tyler, and R. W. Boyd, "Influence of atmospheric turbulence on the propagation of quantum states of light carrying orbital angular momentum," *Opt. Lett.* **34**, 142–144 (2009).
6. R. A. McClatchey and J. E. A. Selby, *Atmospheric Attenuation of Laser Radiation from 0.76 to 31.25 μm* (Environmental Research Paper 460, Air Force Cambridge Research Laboratories, 3 January 1974).
